# Mapping the global landscape of biofilm-associated antimicrobial resistance (1992–2025)

**DOI:** 10.1016/j.bioflm.2026.100358

**Published:** 2026-03-10

**Authors:** Caixia Tan, Jie Wang, Anhua Wu, Chunhui Li

**Affiliations:** a Infection Control Center, Xiangya Hospital, Central South University, Changsha, Hunan Province, 410008, China; b National Clinical Research Center for Geriatric Disorders (XiangYa Hospital), Changsha, Hunan Province, 410008, China; c Superbugs and Multidrug Resistant Microbes Infection Control Research Center, Changsha, Hunan Province, 410008, China

**Keywords:** Antimicrobial resistance (AMR), Biofilm, Bibliometric analysis, *Anti*-biofilm therapeutics, Global health

## Abstract

Antimicrobial resistance (AMR) is a great global health threat, with biofilm formation recognized as a key microbial survival strategy that promotes persistence and recurrent infections. Despite growing mechanistic insights, research on biofilm-associated AMR ((biofilm-AMR)) remains fragmented, limiting the development of broadly effective interventions. To address this gap, we conducted a bibliometric analysis of 17,198 publications from the Web of Science Core Collection (retrieved November 4, 2025) using Bibliometrix–Biblioshiny, CiteSpace, and Excel. Publication output accelerated sharply after 2015, alongside diversification of research themes and increasing interdisciplinary integration. High-output countries and institutions, predominantly in Asia, Latin America, and the Middle East, emphasize natural products, nanomaterials, anti-quorum-sensing strategies, and plant-derived antimicrobials, reflecting application-oriented approaches. In contrast, high-impact contributors in North America and Europe focus on clinical microbiology, resistance mechanisms, pathogen genomics, and hospital infection control, highlighting translational relevance. Trend analyses reveal key topics including quorum sensing, persister-cell biology, multidrug-resistant pathogens, and innovative interventions such as phage therapy, antimicrobial peptides, CRISPR-based antimicrobials, and nanotechnology-enabled drug delivery. Emerging directions include environmental AMR, One Health perspectives, and computational modeling. Despite mechanistic and technological advances, translational barriers persist due to biofilm heterogeneity and model limitations. Promoting interdisciplinary collaboration that integrates basic research, clinical microbiology, materials science, and computational approaches will be essential to accelerate clinical translation and develop effective, globally relevant strategies against AMR.

## 1 Introduction

Antimicrobial resistance (AMR) is a significant global health challenge that threatens the effectiveness of modern medicine [1]. The World Health Organization (WHO) has identified AMR as one of the top ten public health threats, contributing to an estimated 4.95 million deaths annually and undermining the success of routine surgical procedures, cancer therapies, and critical care interventions [2]. Beyond the human impact, AMR imposes a substantial economic burden. Projections estimate that AMR may incur an estimated US$1 trillion in additional healthcare expenditure by 2050, along with annual global Gross Domestic Product (GDP) losses ranging from US$1 trillion to US$3.4 trillion by 2030 [3]. While ongoing efforts in antibiotic stewardship and novel drug development have helped mitigate the pace of resistance emergence, they remain insufficient to reverse the current global trend. As the efficacy of existing antibiotics continues to be compromised by the spread of resistant pathogens, there is an urgent need to deepen our understanding of microbial survival strategies, particularly those underlying resistance, persistence, and treatment failure [4].

One of the most critical of these microbial survival strategies is biofilm formation, which allows microorganisms to evade antimicrobial treatments and persist in hostile environments [5]. Biofilms are structured microbial communities encased in a self-produced extracellular polymeric substance (EPS) matrix. They can form on both biotic and abiotic surfaces and confer antimicrobial tolerance levels up to 1000-fold higher than those of planktonic cells [6,7].This remarkable tolerance results from multiple synergistic mechanisms, including physical protection by the EPS matrix, metabolic heterogeneity, horizontal gene transfer (HGT), and the presence of dormant persister cells [8]. Remarkably, biofilms are implicated in over 80% of chronic and device-associated infections worldwide [9]. These include infections associated with indwelling medical devices, urinary tract infections (UTI), burn wounds, and chronic skin lesions, all of which are notoriously difficult to treat [10]. Effective management often necessitates aggressive interventions such as surgical removal of infected implants or debridement of necrotic tissue. These procedures are invasive, expensive, and frequently associated with prolonged hospitalization, increased risk of complications, and significant strain on healthcare resources. Moreover, biofilm-associated infections commonly act as reservoirs for multidrug-resistant organisms (MDROs), contributing to treatment failure and recurrent infections.

In recent years, significant progress has been made in understanding biofilm-associated AMR (biofilm-AMR), particularly in elucidating key molecular pathways such as quorum sensing and cyclic-di-GMP signaling. Advances have also been achieved in identifying mechanisms of drug tolerance, such as persister cell formation and metabolic heterogeneity, as well as in the development of sophisticated in vitro and in vivo models [[11], [12], [13]]. Beyond these foundational studies, recent research has explored novel anti-biofilm strategies, including the use of nanomaterials, reduced graphene oxide sheets, photodynamic therapies, and ect., which have shown promise in disrupting multi-drug resistant biofilms and combating antimicrobial resistance [[14], [15], [16]]. Despite these advancements, the research landscape remains fragmented, primarily focused on specific pathogens, individual resistance mechanisms, or particular therapeutic approaches, which limits the development of broadly applicable prevention and treatment strategies. Consequently, a comprehensive, global understanding of the field's evolution, dominant research themes, and critical knowledge gaps is essential. Although previous reviews have deepened our understanding of biofilm-AMR interactions—such as how biofilms exacerbate resistance, exploring anti-biofilm therapeutic strategies, and addressing diagnostic gaps in biofilm-AMR susceptibility testing—these efforts have been constrained by qualitative syntheses of selected studies [[17], [18], [19]].

To address this gap, our study conducted the first comprehensive bibliometric analysis of global biofilm-AMR research published between 1992 and 2025. Unlike traditional reviews, bibliometric methods allow for large-scale, quantitative analysis of publication trends, citation impact, and thematic trajectories, providing macroscopic insights that are inaccessible to conventional syntheses. Specifically, this study aims to: (i) trace the temporal and geographical growth of the field; (ii) identify the leading authors, institutions, and countries shaping the research landscape; (iii) compare differences in research emphases between the top ten most productive countries/institutions/authors and those with the highest average citations (AC); (iv) delineate dominant and emerging thematic areas; and (v) highlight underexplored topics and translational barriers that hinder clinical application. By providing a time-resolved, data-driven overview, this work offers researchers, clinicians, and policymakers an evidence-based framework to guide future priorities, foster strategic collaborations, and inform interventions against biofilm-driven AMR.

## 2 Methods

### 2.1 Data source and search strategy

All relevant publications were retrieved from the Web of Science Core Collection (WoSCC) on November 4, 2025. The key search terms are summarized as follows: the AMR concept group included terms such as “multidrug resistant∗", “MDR”, “antibiotic resistance”, “antimicrobial resistance”, “antifungal resistance”, and related variants; the biofilm concept group included the terms “biofilm” and “biofilms”. These two concept groups were combined using the “AND” operator. The detailed list of search terms and operators was shown in Table S1.

### 2.2 Data extraction and processing

The initial dataset was exported from WoSCC in plain text format and imported into Bibliometrix via the Biblioshiny interface, an open-source bibliometric analysis package implemented in RStudio v4.2.2. Core bibliometric indicators were extracted, including annual publication output, citation metrics, keyword clustering, and reference bursts. To provide a comprehensive assessment of publication impact, three core citation metrics were used, each chosen to suit specific analytical purposes, ensuring both accuracy and rigor. Total citations (TC) were used to measure the overall cumulative impact of publications or research entities (e.g., countries, institutions, journals), offering a straightforward reflection of the scholarly attention received. AC was applied to compare the relative impact across different research entities (e.g., high-performing countries, institutions, and journals). This metric was specifically chosen for its ability to normalize citation impact by the total number of publications, thereby mitigating potential bias from differences in publication volume. Although citation lag—particularly for recently published articles—can temporarily reduce AC values, AC remains a robust and widely accepted metric for comparative impact assessment, as it focuses on relative rather than absolute citation performance. This ensures that, even with citation lag, the comparative relationship between research entities (e.g., identifying which institutions or countries have higher per-publication impact) remains meaningful. In addition, mean citations per year (MeanTCperYear) was used to assess the temporal dynamics of citation impact, accounting for the age of publications and mitigating the potential influence of citation lag, especially when comparing the impact of publications from different years. For transparency, self-citations were explicitly retained in the citation analysis. Data cleaning, statistical analyses, and the creation of descriptive tables and trend charts were performed using Microsoft Excel 2021 and RStudio v4.2.2. Data cleaning included standardizing country names (e.g., “USA” to “United States of America” and “UK” to “United Kingdom”) and consolidating synonyms or alternative terms for keywords (e.g., “bacteriophage” and “bacteriophage (phage)" unified as “phage”). To identify and visualize citation bursts, CiteSpace v6.3.1 [20] was used.

## 3 Results

### 3.1 Temporal trends in publications

**Fig. 1:**
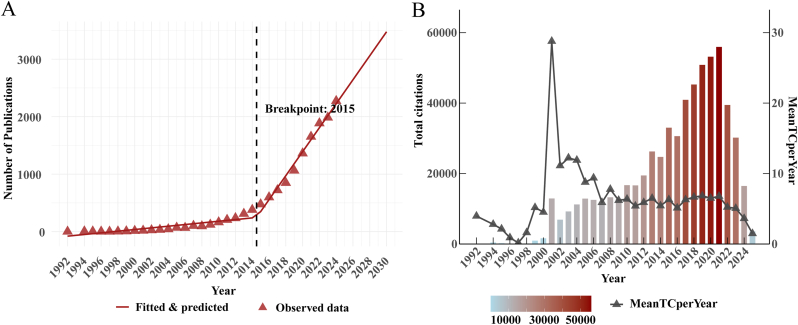
Annual output and citation dynamics of biofilm-associated antimicrobial resistance (biofilm–AMR) research. (A). Annual publication output and growth trend in biofilm–AMR research (1996–2024). The red line represents the fitted and predicted values, while the red triangles represent the observed data. The breakpoint in 2015 indicates a sharp increase in publication output. (B) Temporal patterns of total citations (TC) (bars) and mean citations per year (MeanTCperYear)(grey trace) from 1992 to 2024, with bar colors ranging from light blue (low) to dark red (high) indicating citation counts.(For interpretation of the references to color in this figure legend, the reader is referred to the Web version of this article.)

After applying filters based on publication year, document type, and language type, a total of 17198 articles were included for further analysis (Table S2). To assess temporal trends in research activity, a piecewise linear regression model was applied to annual publication data from 1992 to 2024 (Fig. 1A). The 2025 data were excluded due to their incomplete nature, as publications for the year are still ongoing and may not fully reflect the final trend. The model revealed a statistically significant breakpoint around 2015, indicating a notable shift in the field's growth trajectory. Before 2015, the annual growth in publications was relatively modest, with an estimated slope of 14.41 publications per year. However, after 2015, the rate of publication saw a sharp acceleration, with the growth rate increasing significantly to 208.6 publications per year. This dramatic rise suggests a surge in research activity related to biofilms-AMR, highlighting a growing interest and investment in these areas.

### 3.2 Temporal patterns in citation impact

To evaluate the evolving scholarly influence of biofilm–AMR research, we analyzed annual TC and MeanTCperYear, a metric that normalizes citations by the number of years since publication. As shown in Fig. 1B, citation activity was highly skewed in the early period, driven by a small number of seminal papers. Notably, despite low publication volume, 1999 and 2001 showed marked citation spikes (TC: 979 and 12952, respectively), highlighting the significant influence of early foundational work. TC rose substantially after 2010 and reached a second surge during 2015–2019, paralleling the rapid post-2015 expansion of publications and increased cross-disciplinary engagement. However, MeanTCperYear declined after 2019, a typical trend for more recent publications (2022–2024), due to citation lag rather than reduced scientific relevance. In summary, these patterns emphasize the lasting influence of early landmark studies and the accelerated scholarly attention biofilm and AMR research received post-2015. Although recent publications show a citation lag, the overall trajectory remains upward.

### 3.3 Analysis of prolific countries and institutions

#### 3.3.1 Analysis of the top ten national contributions

**Fig. 2:**
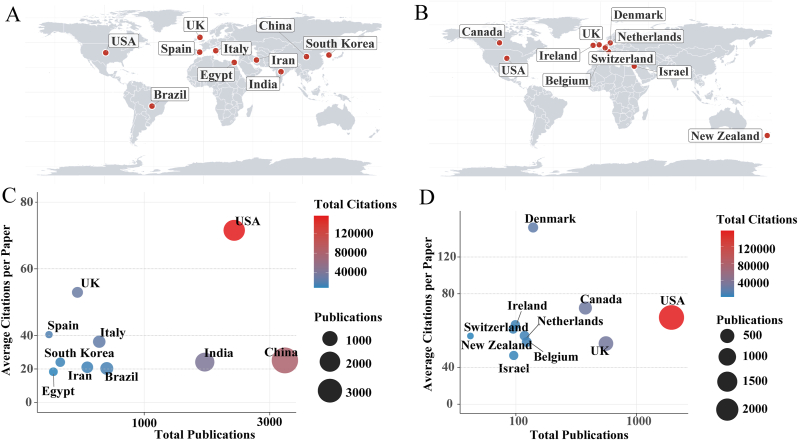
Global landscape of leading countries in biofilm-associated AMR research. (A-B) Geographical distribution of the top 10 countries/regions ranked by total number of publications (A) and by average citations (AC) (B). (C-D) Publication performance of the top 10 countries/regions, ranked by total publications (C) and by AC (D). In both panels, the x-axis shows the total number of publications, the y-axis represents AC, the circle size corresponds to the total number of publications, and the color gradient (darker red = higher) indicates total citations. (For interpretation of the references to color in this figure legend, the reader is referred to the Web version of this article.)

As shown in Fig. 2 and Table S3, substantial geographic and thematic differences were observed between the top ten corresponding countries ranked by publication counts and those ranked by AC. High-output countries such as China (3407 publications, AC = 23.76), India (1699 publications, AC = 24.10), and Brazil (721 publications, AC = 20.32) are primarily located in Asia and Latin America. This pattern aligns with their prominence in various scientific fields, where these countries consistently rank among the highest in publication output [21,22]. The substantial research activity in these regions may be attributed to factors such as significant investments in scientific research, large and growing research networks, and an increasing emphasis on health-related studies [23]. These nations, despite their large research output, exhibit relatively modest citation impact. Their work is characterized by high-throughput experimental models and large-scale applied studies, particularly in AMR gene profiling, natural product–based anti-biofilm strategies, and environmental resistome assessments. Traditional knowledge systems also shape research priorities. China frequently investigates herbal medicines (e.g., Scutellaria baicalensis, berberine) for their anti-biofilm and anti-AMR activities, whereas India focuses on plant-derived antimicrobial agents (e.g., curcumin) with demonstrated roles in inhibiting biofilm formation, disrupting EPS, or enhancing antibiotic susceptibility [[24], [25], [26]].

In contrast, countries with high citation impact—such as Denmark (139 publications, AC = 136.03), New Zealand (42 publications, AC = 57.07), and Canada (377 publications, AC = 72.26)—are primarily located in Europe and North America. Their research emphasizes specialized topics and advanced methodologies, exemplified by Canada's development of microfluidic biofilm models and Denmark's longitudinal investigations into biofilm evolution in chronic infections, including cystic fibrosis [27,28]. Denmark and Belgium have made notable contributions to elucidating the molecular mechanisms underlying biofilm-associated resistance, particularly quorum sensing and toxin–antitoxin regulatory systems [29,30]. Israel (96 publications, AC = 46.48) stands out for its innovative work on alternative therapeutic approaches, such as phage therapy, antimicrobial peptides, and nanotechnology-based strategies for biofilm control [[31], [32], [33]].

Notably, both the United States of America (USA) (2198 publications, AC = 71.54) and the United Kingdom (UK) (558 publications, AC = 52.96) appear among the top ten in both publication volume and citation impact, reflecting a balanced and comprehensive research strategy. In the USA, research spans from elucidating the mechanisms underlying biofilm tolerance to advancing translational technologies, including phage therapy, CRISPR-enabled antimicrobial strategies, and novel drug delivery systems [[34], [35], [36], [37]]. The UK emphasizes clinically relevant models—such as chronic wound and catheter-associated infections—and has advanced biofilm research–host interactions and natural product–based control strategies [[38], [39], [40]]. Collectively, these patterns highlight how different countries contribute in complementary ways to biofilm–AMR research. High-output countries mainly lead large-scale and application-oriented studies supported by extensive research networks, whereas high-impact countries more often focus on innovative, mechanistic, and technology-intensive work. Together, these efforts advance a more comprehensive understanding of biofilm-AMR.

#### 3.3.2 Analysis of top ten institutional contributions

To assess how national AMR strategies are reflected in institutional research activity, we analyzed the top ten corresponding institutions ranked by publication volume and academic influence (Table S3). These leading institutions demonstrate considerable geographic diversity, with many located in developing countries such as India, Iran, and Egypt. These universities and research centers often draw on local biological resources to advance natural product–based antimicrobial approaches. Representative institutions—such as the Chinese Academy of Sciences, Alagappa University, and King Saud University—frequently investigate plant-derived extracts, anti-quorum-sensing and anti-virulence strategies, as well as green-synthesized antimicrobial nanoparticles [[41], [42], [43]]. Additionally, these institutions maintain extensive international collaboration networks, which help mitigate regional resource constraints. Specifically, the study collaboration rates for the Chinese Academy of Sciences, Alagappa University, and King Saud University were 72.8%, 71.15%, and 57.89%, respectively. For instance, Chinese Academy of Sciences often collaborates with prominent Chinese universities, such as Shanghai Jiao Tong University and Jilin University. Alagappa University partners with Bharathidasan University (India) and Shanghai Jiao Tong University (China), while King Saud University works primarily with Aligarh Muslim University (India), Qassim University, and other national universities in Saudi Arabia. These collaborative efforts highlight their active involvement in the global AMR research network and underscore their role in fostering interdisciplinary and international cooperation in the fields of biofilms and AMR. Overall, their work reflects a clear orientation toward application-focused basic research, with an emphasis on identifying new anti-biofilm compounds and elucidating inhibitory mechanisms. By contrast, high-impact institutions—primarily located in North America and Europe—tend to concentrate on clinically driven themes, including antimicrobial resistance mechanisms, hospital infection control, and pathogen genomic evolution. Institutions such as Dartmouth Medical School, Tufts University, and Denmark's Rigshospitalet routinely analyze resistance patterns in clinical isolates, conduct antibiotic susceptibility surveillance, and trace the spread of resistance genes [[44], [45], [46]]. Their research aligns closely with clinical priorities and global AMR governance agendas, enabling these institutions to generate substantial public health impact and achieve strong citation performance. Overall, the institutional-level results parallel the national trends, showing that both large-scale output and high-impact, innovation-oriented research coexist within the field.

### 3.4 Research themes of influential authors

**Fig. 3:**
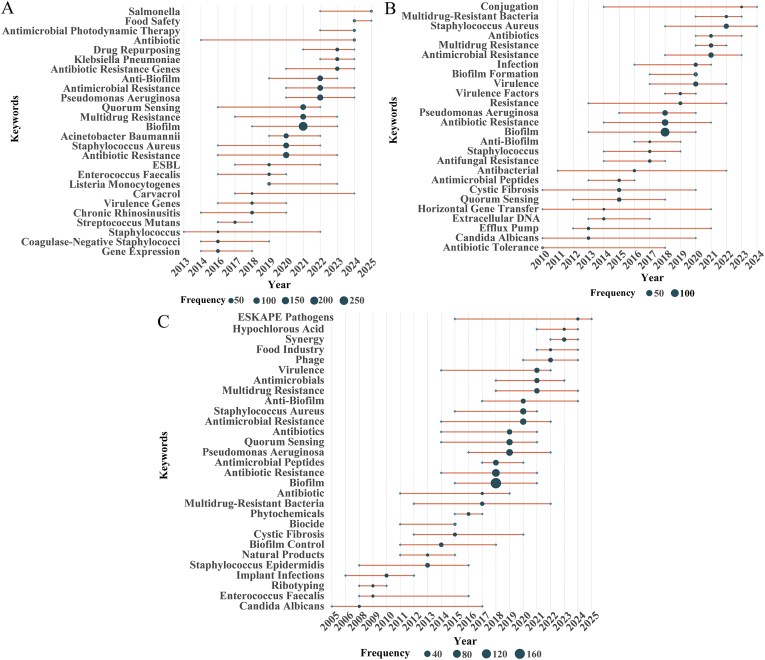
Comparative thematic evolution of author clusters in biofilm-associated antimicrobial resistance research. (A) Top 10% of authors ranked by publication output; (B) Top 10% of authors ranked by average citations; (C) Authors overlapping in both productivity and citation impact groups. In three panels, the size of the circles represents the frequency of the keywords, while the horizontal lines indicate the time span of keyword occurrence. The position of the circles along the x-axis corresponds to the middle year of the keyword's usage.

To elucidate how different contributor groups shape the scientific landscape of biofilm and AMR research, we first identified the top 10% of authors by publication output (high-productive authors) and the top 10% of authors by AC (high-impact authors), as well as the subset of individuals who belong to both categories. We then compared the topic evolution across these three groups (Fig. 3). High-productive authors exhibit a dynamic shift in focus, adapting to emerging clinical challenges (Fig. 3A). From 2013 to 2016, their research primarily concentrated on gene expression and coagulase-negative *staphylococci*, laying the groundwork for pathogenic microbiology. Between 2017 and 2020, their scope expanded to include natural products such as carvacrol, extended-spectrum beta-lactamases (ESBL), *Listeria species*, and virulence-associated genes. This period marked a shift toward investigating natural products and virulence regulation as novel research areas. From 2021 to 2024, their attention turned to *Pseudomonas aeruginosa* (*P. aeruginosa*), AMR, biofilm inhibition, and antibiotic resistance genes. Additionally, they incorporated emerging topics such as drug repurposing, antimicrobial photodynamic therapy, and food safety, reflecting a transition from studying individual pathogens to integrating diverse technological solutions (Fig. 3A).

The research trajectory of high-impact authors follows a logical progression from foundational to applied research. Between 2010 and 2014, their focus was on core resistance mechanisms—such as efflux pumps and HGT—which provided key insights into the genetic basis of drug resistance. From 2015 to 2019, their research expanded to include broader concepts like quorum sensing, antimicrobial peptides, and biofilm formation, exploring how these factors contribute to resistance through intercellular signaling and biofilm-mediated mechanisms. Between 2020 and 2024, their work increasingly focused on pressing clinical issues, including antimicrobial and multidrug resistance, *Methicillin-resistant Staphylococcus aureus* (MRSA), and plasmid-mediated resistance transfer. This shift represented a move from basic research to the development of practical strategies for combating antibiotic-resistant infections (Fig. 3B).

Authors who were both highly productive and impactful followed a pattern of deepening expertise before expanding their focus to emerging research topics. From 2010 to 2014, their research concentrated on core resistance mechanisms, such as efflux pumps and HGT, while also establishing chronic infection models involving *Candida albicans* (*C. albicans*) and implant-related infections. This dual focus on mechanisms and clinical relevance provided a strong foundation for their work. Between 2015 and 2020, they broadened their research to include quorum sensing, antimicrobial peptides, and biofilm control, building a comprehensive framework for addressing antimicrobial resistance. From 2021 to 2025, they rapidly incorporated emerging areas like phage therapy, synergistic treatments, and ESKAPE pathogens, bringing these topics to the forefront of both academic research and clinical practice (Fig. 3C).

### 3.5 Analysis of journals

**Fig. 4:**
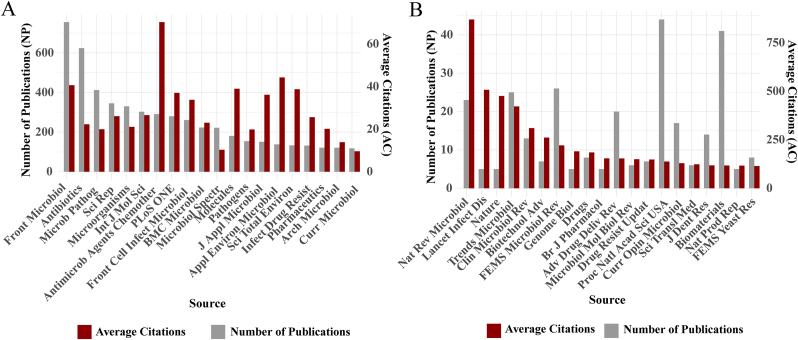
Top 20 journals in biofilm–AMR research ranked by publication count and citation impact. (A) Top 20 journals ranked by total number of publications (NP). (B) Top 20 journals ranked by average citations per article (AC). In both panels, the left y-axis indicates the number of publications (grey bars), and the right y-axis indicates the AC (red bars). Journals are presented in descending order based on the primary ranking metric (NP in A, AC in B). (For interpretation of the references to color in this figure legend, the reader is referred to the Web version of this article.)

To characterize journal-level publication patterns, we compared the top 20 journals by publication volume and by AC (Fig. 4; Table S4). The analysis revealed a noticeable gap between productivity and citation influence. High-output journals—such as *Frontiers in Microbiology* (755 articles, AC = 40.6) and *Antibiotics* (624 articles, AC = 22.3)—serve as major outlets for biofilm–AMR studies, but achieved only moderate citation performance. In contrast, several journals with relatively small publication counts—*Nature Reviews Microbiology* (23 articles, AC = 870.4), Th*e Lancet Infectious Diseases* (5 articles, AC = 507.8), Nature (5 articles, AC = 476), and *Trends in Microbiology* (25 articles, AC = 422.2)—show remarkably high citation averages. This pattern reflects the nature of the work they publish, which often includes authoritative reviews, conceptual pieces, or perspective articles that draw considerable attention. Similar trends were observed for interdisciplinary or review-oriented journals such as *Biotechnology Advances*, *Clinical Microbiology Reviews*, and *Drug Resistance Updates*, where manuscripts frequently bridge microbiology with areas including nanotechnology, biomaterials, drug development, or translational AMR research. Notably, multidisciplinary journals like *Science of the Total Environment* also show lower AC values (133 articles, AC = 38.8), likely because biofilm–AMR studies account for only a small fraction of their overall scope, reducing field-specific visibility. Taken together, the influence of a journal in the biofilm–AMR field depends on more than its output or impact factor. Selectivity, topical focus, and alignment with emerging research directions all play important roles. High-output journals contribute to steady knowledge accumulation, whereas selective or cross-disciplinary venues tend to publish the papers that attract widespread attention.

### 3.6 Analysis of trend topics

#### 3.6.1 Keyword frequency and thematic distribution

**Fig. 5:**
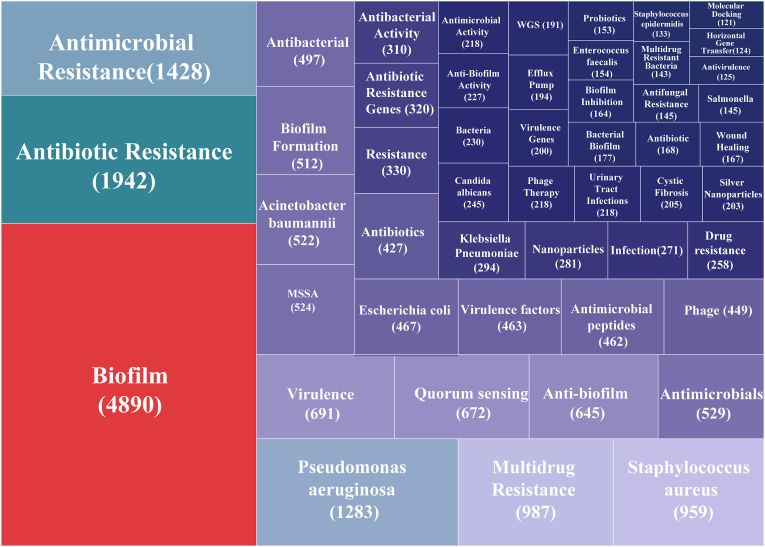
Treemap of the top 50 high-frequency keywords. Rectangle size and values in parentheses represent keyword frequency, with larger rectangles indicating higher frequencies.

To outline the major research focuses within the biofilm–AMR field, we first examined keyword frequency patterns across 17,198 publications. The 50 most frequent keywords were extracted for visualization and thematic interpretation, revealing a highly structured and clinically oriented thematic landscape (Fig. 5). Beyond the core concepts of biofilms and antimicrobial resistance, the consistent prominence of major bacterial pathogens—including *P. aeruginosa*, *Staphylococcus aureus* (*S. aureus*), *Escherichia coli* (*E. coli*), *Acinetobacter baumannii* (*A. baumannii*), *klebsiella pneumoniae* (*K. pneumoniae)* and *Staphylococcus epidermidis* (*S. epidermidis*)—underscores the field's sustained clinical relevance. Mechanistic terms such as quorum sensing, efflux pumps, HGT, and whole-genome sequencing reflect continued efforts to elucidate regulatory and genetic contributors to biofilm-associated tolerance. Simultaneously, the emergence of therapy-associated keywords, including antimicrobial peptides, phage therapy, nanoparticles, and silver-based materials, highlights growing interest in alternative or adjunctive strategies to overcome recalcitrant biofilm infections. The prominence of fungal pathogens (*C. albicans*, *C. auris*) and clinical contexts such as UTI, cystic fibrosis, and wound healing further illustrates the broad disease spectrum involved in biofilm–AMR research.

#### 3.6.2 Keyword cluster structure

**Fig. 6:**
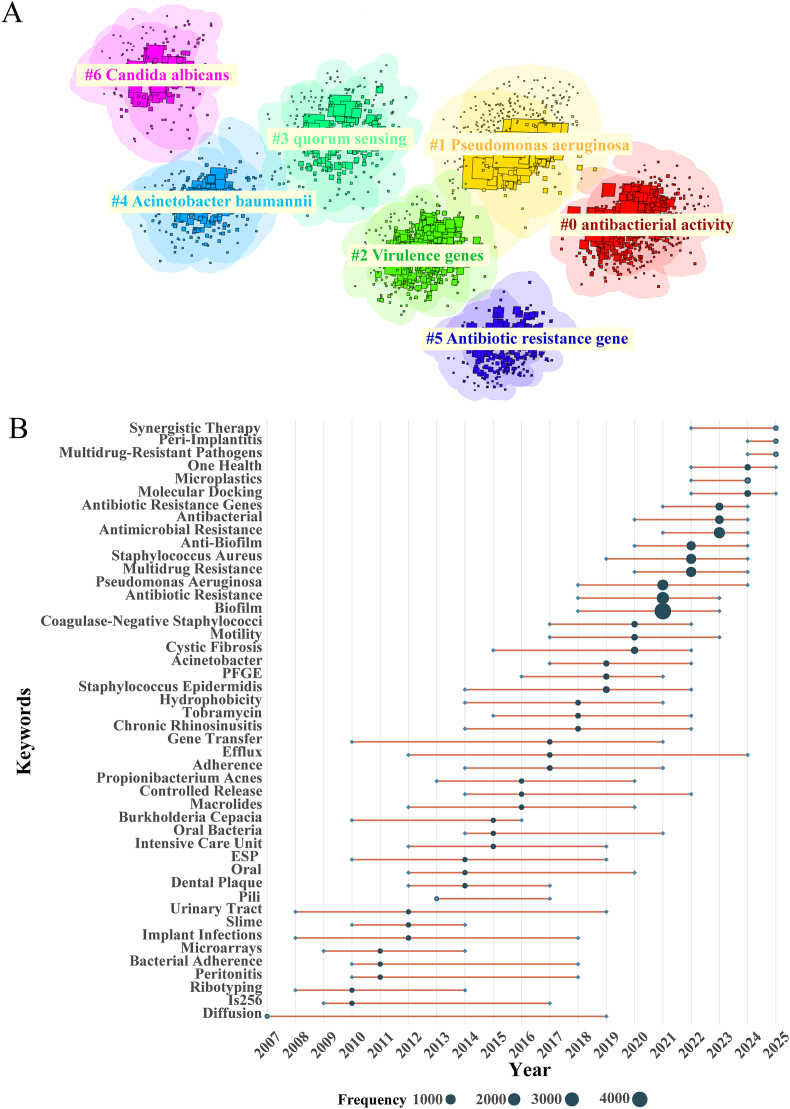
Trend topics in the biofilm-associated antimicrobial resistance research field (1994-2023). (A) CiteSpace-based clustering visualization of keywords. Nodes represent individual keywords, colors denote distinct clusters (#0–#6), with smaller cluster numbers indicating larger cluster sizes. (B) A time-based keyword trajectory plot. Circle size indicates the relative frequency of each term, while the orange segment marks the period during which the keyword remained prominent. The position of the circles along the x-axis corresponds to the middle year of the keyword's usage. (For interpretation of the references to color in this figure legend, the reader is referred to the Web version of this article.)

To elucidate the relationships among these thematic elements, we applied log-likelihood ratio (LLR) clustering in CiteSpace, which yielded seven distinct thematic clusters (Fig. 6A). Cluster 0—the largest—primarily comprises research on antimicrobial and nanomaterial-based interventions, underscoring the central role of therapeutic development. Cluster 1 groups major multidrug-resistant bacterial pathogens, reflecting persistent clinical challenges. Clusters 2 and 3 concentrate on mechanistic investigations, particularly virulence regulation, host-associated bacteria, and quorum-sensing networks. Cluster 4 links multidrug-resistant Gram-negative organisms with bacteriophage-based strategies, aligning with growing interest in phage therapy as a potential anti-biofilm modality. Clusters 5 and 6 address the environmental dissemination of resistance and fungal biofilm biology, indicating increasing attention to One Health perspectives and cross-kingdom interactions. Together, these clusters demonstrate the field's multidimensional nature, integrating mechanistic, clinical, therapeutic, and environmental research axes.

#### 3.6.3 Temporal evolution of research themes

To trace how research priorities have shifted over time, we analyzed the temporal evolution of major trend topics (Fig. 6B). Early studies (2007–2013) focused primarily on foundational aspects of biofilm formation and antimicrobial tolerance. Between 2014 and 2018, research gradually transitioned toward clinical contexts, particularly biofilm-associated infections and specific resistance mechanisms such as efflux activity. From 2019 to 2024, the field expanded toward therapeutic innovation, with notable growth in studies exploring alternative antimicrobials, anti-biofilm agents, and phage-based interventions. The most recent period (2024–2025) introduced emerging themes, including microplastics and molecular docking, reflecting increased attention to environmental exposure pathways and computational design tools. Collectively, these temporal dynamics illustrate a shift from mechanism-focused inquiry toward clinically grounded, intervention-oriented, and environmentally informed research, highlighting the field's continued diversification.

### 3.7 Top ten most original research and review articles

To gain a comprehensive understanding of the influence of biofilm-AMR research, we separately analyzed the top 10 most-cited original research articles and review articles. This approach ensures an appropriate assessment of the distinct roles these types of publications play in advancing the field—empirical studies contribute novel data, while reviews synthesize and integrate existing knowledge. The ten most-cited original research articles focused on the core mechanisms of biofilm-AMR, with global citation counts ranging from 671 to 1270 (Table 1). Early studies published between 2001 and 2004 laid the foundational groundwork for understanding biofilm development, structure, and drug resistance in major pathogens, including *C. albicans*, *P. aeruginosa*, *Vibrio cholerae*, and *E. coli*. For instance, Chandra J et al. elucidated biofilm development, structure, and drug resistance in *C. albicans*, while Mah TF et al. revealed the genetic basis of *P. aeruginosa* biofilm antibiotic resistance [47,48]. Drenkard et al. further confirmed the link between *Pseudomonas* biofilm formation, antibiotic resistance, and phenotypic variation, offering updated insights into these core mechanisms [49]. Studies from 2005 to 2006 expanded the field by investigating the genetic adaptation of *P. aeruginosa* during chronic respiratory infection, genome evolution in methicillin-resistant *staphylococci*, and in vivo biofilm detection in clinical infections, highlighting the translational relevance of basic biofilm research [[50], [51], [52]].

**Table 1:** Top ten globally most cited original research articles in biofilm-associated AMR research.

Title	First author	Published time	Journal	DOI	Global Citations
Biofilm formation by the fungal pathogen Candida albicans: development, architecture, and drug resistance	CHANDRA J	2001	Journal of Bacteriology	10.1128/JB.183.18.5385-5394.2001	1270
Genetic adaptation by Pseudomonas aeruginosa to the airways of cystic fibrosis patients	SMITH EE	2006	Proceedings of the National Academy of Sciences of the United States of America	10.1073/pnas.0602138103	1080
A genetic basis for Pseudomonas aeruginosa biofilm antibiotic resistance	MAH TF	2003	Nature	10.1038/nature02122	920
Insights on evolution of virulence and resistance from the complete genome analysis of an early methicillin-resistant Staphylococcus aureus strain and a biofilm-producing methicillin-resistant Staphylococcus epidermidis strain	GILL SR	2005	Journal of Bacteriology	10.1128/JB.187.7.2426-2438.2005	833
Inhibition of quorum sensing in Pseudomonas aeruginosa biofilm bacteria by a halogenated furanone compound	HENTZER M	2002	Microbiology (Society for General Microbiology)	10.1099/00221287-148-1-87	784
Pseudomonas biofilm formation and antibiotic resistance are linked to phenotypic variation	DRENKARD E	2002	Nature	10.1038/416740a	762
Biofilms and planktonic cells of Pseudomonas aeruginosa have similar resistance to killing by antimicrobials	SPOERING AL	2001	Journal of Bacteriology	10.1128/JB.183.23.6746-6751.2001	720
Direct detection of bacterial biofilms on the middle-ear mucosa of children with chronic otitis media	HALL-STOODLEY L	2006	JAMA: The Journal of the American Medical Association	10.1001/jama.296.2.202	706
Quorum sensing controls biofilm formation in Vibrio cholerae	HAMMER BK	2003	Molecular Microbiology	10.1046/j.1365-2958.2003.03688.x	693
Specialized persister cells and the mechanism of multidrug tolerance in Escherichia coli	KEREN I	2004	Journal of Bacteriology	10.1128/JB.186.24.8172-8180.2004	671

In contrast, the top 10 most-cited review articles had significantly higher citation counts, ranging from 1484 to 5389, reflecting their critical role in summarizing, integrating, and disseminating key advancements across the biofilm-AMR field (Table 2). Consistent with original research, early influential reviews published between 2001 and 2004 formalized core theories of biofilm resistance. Stewart et al. provided an overview of antibiotic resistance in bacterial biofilms, Mah et al. discussed the mechanisms underlying biofilm resistance to antimicrobial agents, and Davies et al. offered a comprehensive understanding of biofilm resistance to antibacterial agents [19,34,53]. From 2007 to 2010, highly cited reviews expanded to address specialized topics, including persister cell dormancy and its association with infectious diseases, physiological heterogeneity in biofilms, and a systematic update on antibiotic resistance in bacterial biofilms [[54], [55], [56]]. After 2015, review articles increasingly focused on clinical and translational perspectives. Flores-Mireles et al. explored UTI, focusing on the role of biofilm formation in infection, epidemiology, and treatment, while Pang et al. emphasized antibiotic resistance in *P. aeruginosa* and alternative therapeutic strategies [57,58]. Among these, Hall-Stoodley et al. published the most highly cited review, which bridged bacterial biofilms from the natural environment to infectious diseases and became a landmark reference for integrating environmental and clinical biofilm research [59].

**Table 2:** Top ten globally most cited review articles in biofilm-associated AMR research.

Title	First author	Published time	Journal	DOI	Global Citations
Bacterial biofilms: from the natural environment to infectious diseases	HALL-STOODLEY L	2004	Nature Reviews Microbiology	10.1038/nrmicro821	5389
Antibiotic resistance of bacteria in biofilms	STEWART PS	2001	The Lancet	10.1016/S0140-6736(01)05321-1	3491
Mechanisms of biofilm resistance to antimicrobial agents	MAH TFC	2001	Trends in Microbiology	10.1016/S0966-842X(00)01913-2	2932
Urinary tract infections: epidemiology, mechanisms of infection and treatment options	FLORES-MIRELES AL	2015	Nature Reviews Microbiology	10.1038/nrmicro3432	2504
Antibiotic resistance of bacterial biofilms	HOIBY N	2010	International Journal of Antimicrobial Agents	10.1016/j.ijantimicag.2009.12.011	2418
Understanding biofilm resistance to antibacterial agents	DAVIES D	2003	Nature Reviews Drug Discovery	10.1038/nrd1008	2245
Physiological heterogeneity in biofilm	STEWART PS	2008	Nature Reviews Microbiology	10.1038/nrmicro1838	1739
Persister cells, dormancy and infectious disease	LEWIS K	2007	Nature Reviews Microbiology	10.1038/nrmicro1557	1533
Riddle of biofilm resistance	LEWIS K	2001	Antimicrobial Agents and Chemotherapy	10.1128/AAC.45.4.999-1007.2001	1495
Antibiotic resistance in Pseudomonas aeruginosa: mechanisms and alternative therapeutic strategies	PANG Z	2019	Biotechnology Advances	10.1016/j.biotechadv.2018.11.013	1484

Together, these highly cited original research and review articles illustrate a clear progression from foundational mechanistic studies to clinically oriented and translational research, paralleling global concerns about biofilm-AMR. The marked difference in citation counts between original research and review articles underscores the need for stratified analysis when evaluating publication impact—original studies lay the empirical groundwork, while reviews synthesize knowledge and guide future research directions, with both playing indispensable roles in advancing the field.

### 3.8 References exhibiting the strongest citation bursts

Analysis of the 25 co-cited references with the strongest citation bursts revealed a clear temporal progression in research priorities (Fig. S1). The earliest influential studies, including those by Drenkard (2002) and Høiby (2010), established the clinical significance of biofilm-associated infections and elucidated the physiological and structural bases of persistent, drug-refractory phenotypes [49,56]. Between 2014 and 2020, research attention shifted toward conceptual consolidation and mechanistic depth. Magiorakos's MDR classification system (2014–2017) provided a unified framework for interpreting resistance patterns[60], while studies by de la Fuente-Núñez, Lebeaux, Blair, and Olsen (2013–2020) advanced understanding of the molecular basis of biofilm-associated tolerance, highlighting efflux regulation, stress-response activation, and matrix-mediated protection [[61], [62], [63]]. In the late 2010s and early 2020s, the field entered a more translational phase. Research led by Koo and Hall (2017–2022) exemplified this transition by extending mechanistic insights toward therapeutic innovation, focusing on quorum-sensing disruption, matrix-targeting approaches, and anti-biofilm intervention strategies [64,65]. From approximately 2018 onward, research priorities increasingly reflected global AMR challenges. Roy's work (2018–2023) expanded the scope of anti-biofilm therapeutics, while Tacconelli's analyses provided an authoritative basis for pathogen prioritization and antibiotic stewardship, linking biofilm science with global public health strategies [66,67]. Since 2022, research directions have diversified further. Studies by Ciofu, Sauer, and Qin (2022) deepened investigations into tolerance pathways, biofilm life-cycle transitions, and virulence regulation, whereas Murray's global AMR assessment (2022) highlighted the growing clinical burden associated with biofilm-forming pathogens [11,[68], [69], [70]]. More recently, work by Sharma, continuing into 2025, underscores increasing concern over the expanding threat posed by biofilm-mediated antimicrobial resistance [5]. Collectively, these citation bursts illustrate a progression from early mechanistic foundations to contemporary clinical, ecological, and therapeutic expansions within the biofilm–AMR landscape.

## 4 Discussion

Over the past two decades, biofilm–AMR has evolved from a niche topic to a central focus in infectious disease and therapeutic development. Biofilms are now widely recognized not merely as passive barriers to antimicrobials but as dynamic and adaptive ecosystems that actively contribute to resistance development, persistence, and treatment failure. Our bibliometric analysis of literature from 1992 to 2025 reveals a notable inflection point around 2015, marking a transition from largely descriptive studies toward more mechanistic and translational investigations. This shift mirrors the escalating urgency of the global AMR crisis and increasing awareness that biofilm-related infections are among the main drivers of therapeutic failure.

### 4.1 Global research landscape and publication dynamics

Building on the observed growth of biofilm–AMR research, the global landscape has undergone notable geographic and institutional diversification. High-output countries such as China, India, and Brazil mainly engage in application-oriented studies, leveraging local biological resources, traditional knowledge, and large-scale experimental models. Institutions in these countries, including the Chinese Academy of Sciences and Alagappa University, often emphasize broad empirical investigations, natural product–based antimicrobials, anti-biofilm strategies, and environmental resistome mapping, reflecting responsiveness to local health and ecological challenges. In contrast, countries with higher AC—such as the USA, Denmark, and Canada—focus on mechanistic understanding, advanced methodologies, and translational relevance, exemplified by Dartmouth Medical School and Rigshospitalet. Their work spans molecular-level mechanisms, clinical pathogen surveillance, microfluidic biofilm models, and longitudinal studies of biofilm evolution. These patterns indicate that research impact is influenced not only by output volume but by methodological sophistication, experimental rigor, and alignment with pressing biomedical challenges. Emerging research nations may benefit from fostering investigators who balance breadth with mechanistic depth to enhance qualitative influence alongside quantitative growth.

These global patterns are also reflected at the level of individual contributors. High-productive authors cover a broad spectrum of topics and adapt dynamically to emerging clinical challenges, integrating approaches such as natural products, drug repurposing, and antimicrobial photodynamic therapy. In contrast, high-impact authors concentrate on priority pathogens and translationally relevant mechanisms, including efflux pumps, HGT, quorum sensing, and biofilm-mediated resistance, producing work with greater citation influence. Authors who are both highly productive and highly cited combine these strengths, linking foundational mechanistic studies with clinically oriented and frontier therapeutic strategies, including phage therapy, synergistic treatments, and investigations of ESKAPE pathogens. Collectively, these observations suggest that countries and institutions aiming to enhance research impact could benefit from fostering investigators who balance breadth with mechanistic depth, ensuring that empirical productivity aligns with translational significance and strong scholarly influence.

At the journal level, our results suggest that influence in biofilm–AMR research depends more on thematic relevance, methodological rigor, and cross-disciplinary integration than on publication volume or impact factor. High-output journals, such as *Frontiers in Microbiology* and *Antibiotics*, mainly disseminate application-oriented studies, supporting cumulative knowledge, whereas high-impact journals, including *Nature Reviews Microbiology*, The *Lancet Infectious Diseases*, and *Trends in Microbiology*, as well as cross-disciplinary venues like *Biotechnology Advances* and *Clinical Microbiology Reviews*, attract attention through mechanistic, concept-driven, or translational work. These patterns indicate that maximizing visibility and scholarly impact requires aligning studies with pressing scientific and clinical challenges and leveraging innovative or cross-disciplinary approaches. For emerging research communities, targeting relevant journals and emphasizing translational potential can enhance long-term influence, reinforcing that journal selection and research focus critically shape scholarly impact in biofilm–AMR research.

### 4.2 Key topics and research frontiers

Recent thematic evolution in biofilm–AMR research demonstrates a shift from descriptive studies of biofilm formation toward mechanism-driven, clinically oriented, and technology-integrated paradigms. Analyses of keyword frequency, CiteSpace clustering, trend-topic evolution, and citation bursts reveal several key domains that continue to shape the research field.

#### 4.2.1 Biofilm microenvironments and tolerance mechanisms

A central focus is the structural and biochemical basis of biofilm tolerance, particularly the roles of EPS and microenvironmental heterogeneity. EPS is increasingly recognized as an active biochemical matrix capable of cation chelation, sequestration of certain antibiotics, and modulation of redox and nutrient gradients, thereby creating spatially heterogeneous niches that promote metabolic dormancy, persister-cell formation, and reduced antibiotic susceptibility [71]. Citation bursts from recent studies (e.g., Sauer 2022; Ciofu 2022) highlight growing attention to biofilm life-cycle dynamics and physiological plasticity in pathogens such as *P. aeruginosa* and *S. aureus* [11,68]. These insights underscore the importance of considering biofilm microenvironments when designing antimicrobial strategies and predicting treatment outcomes. Microfluidic platforms and organ-on-a-chip systems are increasingly used to replicate biofilm heterogeneity and evaluate antimicrobial responses under physiologically relevant conditions [72]. To overcome translational gaps, future work should further leverage such microenvironment-mimicking in vitro models for anti-biofilm drug screening and more accurate prediction of in vivo efficacy.

#### 4.2.2 Host–biofilm interactions in chronic infections

The interplay between biofilms and host immunity has become increasingly prominent, particularly in chronic infections such as UTI, lung infections in cystic fibrosis patients, and chronic wounds [73]. Biofilms evade immune responses via altered antigen exposure, neutrophil suppression, or release of immunomodulatory vesicles [74]. Citation bursts (e.g., Lebeaux 2014; Ciofu 2022) highlight a growing focus on host–biofilm interactions and environmentally driven adaptations within chronic infection niches [11,62]. These interactions contribute to persistent inflammation and recalcitrant infections, complicating treatment outcomes. To overcome these clinical challenges, rational, targeted combinatorial approaches are needed that simultaneously disrupt biofilm structure and restore effective host immune function [73,75]. Potential strategies include enhancing neutrophil chemotaxis and bactericidal activity, reversing biofilm-induced immune paralysis, and blocking immune-evasive signaling pathways [76]. Notably, combining immune-modulatory agents with anti-biofilm therapies—including antimicrobial peptides, matrix-degrading enzymes, and membrane-active compounds—can restore immune recognition and clearance, alleviate chronic inflammation, and improve outcomes against drug-resistant biofilm infections [77,78].

#### 4.2.3 Next-generation anti-biofilm therapeutics

Therapeutic innovation represents the most rapidly expanding frontier. Since 2019, clusters 0 and 4 reveal growing exploration of antimicrobial peptides, bacteriophages, CRISPR-based antimicrobials, supramolecular nanomaterials, and hybrid photodynamic–nanoparticle systems. Research increasingly links multidrug-resistant Gram-negative pathogens (e.g., *A. baumannii*, *K. pneumoniae*, *P. aeruginosa*) with phage- and nano-based therapies [[79], [80], [81], [82]]. Correspondingly, citation bursts highlight intensified efforts to disrupt EPS architecture, re-sensitize dormant subpopulations, and develop targeted or stimuli-responsive delivery platforms [11,64,65,83]. Together, these trends signal a clear shift toward translationally oriented research addressing urgent clinical challenges.

#### 4.2.4 Ecological dimensions and One Health integration

The field is also expanding ecologically under One Health frameworks. Keywords such as microplastics, wastewater, environmental resistome, and fungal biofilms underscore growing concern for environmental dissemination of AMR determinants. Post-2020 citation bursts indicate increasing integration of environmental microbiology with clinical AMR prioritization, consistent with evidence that biofilm-associated resistance arises across clinical, agricultural, and environmental ecosystems [[84], [85], [86]]. Together, these insights underscore that effective AMR mitigation requires coordinated, multisectoral strategies spanning human health, agriculture, and environmental systems.

#### 4.2.5 Technological convergence and predictive modeling

Emerging topics in 2024–2025 indicate a convergence of experimental and computational approaches in biofilm research. Microfluidic and organ-on-a-chip platforms are increasingly used to replicate the spatial and physiological heterogeneity of biofilms, while whole-genome sequencing and molecular docking provide insights into host–pathogen interactions and drug–biofilm binding dynamics. Such integration enables predictive modeling of biofilm responses to antimicrobial interventions, facilitating the design of targeted, stimuli-responsive, and precision strategies. Together, these developments illustrate a shift toward multiscale, data-driven research that connects mechanistic understanding with potential translational applications.

### 4.3 Translational barriers and future research directions

Biofilms act as dynamic, adaptive ecosystems that drive AMR, infection persistence, immune evasion, and clinical treatment failure. Despite rapid advances in mechanistic understanding and technological innovation, the translation of biofilm-targeted strategies into clinical practice remains markedly limited. A major obstacle lies in the profound heterogeneity of clinically relevant biofilms, which differ across anatomical sites, host environments, disease states, and microbial community structures [87]. These contextual variations reshape biofilm architecture, EPS composition, nutrient and oxygen gradients, and the frequency of persister-cell formation, resulting in a substantial mismatch between laboratory models and in vivo biofilm behavior [88]. A second translational barrier stems from the lack of standardized, clinically meaningful endpoints for evaluating anti-biofilm efficacy [17]. Current assessments typically rely on biomass reduction, minimum inhibitory concentration (MIC)-based susceptibility metrics, or early biofilm inhibition assays, which insufficiently reflect the clinical determinants of treatment success—such as suppression of persister-cell populations, enhancement of antibiotic susceptibility, disruption of microenvironmental tolerance pathways, or prevention of relapse [[89], [90], [91]]. The absence of harmonized analytical frameworks complicates cross-study comparisons, limits reproducibility, and impedes rational prioritization of therapeutic candidates for clinical development. A third translational barrier involves regulatory and clinical validation gaps. Most anti-biofilm agents remain at the preclinical or experimental stage due to unclear regulatory pathways, insufficiently tailored clinical trial designs, and limited consensus on outcome measures [92]. Moreover, the high cost, complexity, and ethical constraints associated with chronic infection models hinder large-scale clinical translation and commercialization [93].

Looking ahead, bridging these translational gaps will require interdisciplinary integration and innovative modeling approaches. First, prioritize the development and application of host-mimetic models (e.g., cystic fibrosis lung-on-a-chip, urinary tract organoids) that replicate the anatomical, physiological, and microbial features of clinical biofilm niches (e.g., mucus hypersecretion in cystic fibrosis, urine pH in urinary tract infections), ensuring more accurate anti-biofilm drug screening and efficacy evaluation [94].Second, apply multi-omics approaches to identify biofilm-specific biomarkers (e.g., EPS-related proteins, persister cell markers, pro-inflammatory cytokines) that can guide treatment monitoring, predict therapeutic responses, and distinguish between biofilm persistence and infection resolution. [95]. Third, establish a global consensus on standardized anti-biofilm endpoints, such as biofilm dispersal rates, relapse prevention duration (e.g., 30-day post-treatment relapse rate in chronic wounds), and host immune modulation (e.g., reduced neutrophil suppression), to facilitate cross-study comparisons and improve clinical trial design. Fourth, advance combinatorial therapeutic strategies, such as combining immune-modulatory agents (e.g., N-acetylcysteine) with biofilm-targeting therapies (e.g., antimicrobial peptides, matrix-degrading enzymes) to disrupt biofilm integrity, reverse immune suppression, and re-sensitize drug-resistant pathogens to antibiotics [76,77,96]. Finally, addressing regulatory and clinical validation barriers will require closer collaboration among researchers, clinicians, and regulatory bodies to streamline approval processes, refine clinical trial protocols, and reduce the cost and complexity of chronic infection models.

## 5 Conclusion

This bibliometric analysis systematically characterizes the evolution of biofilm-AMR research from 1992 to 2025, revealing a significant inflection point in 2015, when publication growth accelerated sharply. Thematic evolution has shifted toward mechanism-driven, clinically oriented research focused on addressing clinical challenges of biofilm-AMR, including biofilm microenvironments in chronic infections, host-biofilm interactions in refractory infections, and next-generation therapeutics for drug-resistant biofilm infections. However, critical translational gaps remain, including mismatches between simplified in vitro models and clinical biofilm heterogeneity, a lack of standardized endpoints for anti-biofilm efficacy evaluation, and regulatory and clinical validation challenges. Future research should prioritize clinically relevant host-mimetic models, multi-omics-derived biomarkers, standardized clinical endpoints, and combinatorial therapies that disrupt biofilms and restore immune function.

## 6 Limitation

Despite its comprehensive nature, this study is limited by the exclusion of non-English language publications and articles not indexed in major bibliometric databases, which may underrepresent research from certain regions. Additionally, the exclusive use of the WoSCC database may introduce selection bias, as other significant databases, such as PubMed and Scopus, were not considered. However, we chose WoSCC for its broad indexing of high-quality, peer-reviewed journals and its established consistency in citation analysis. Moreover, while the study analyzes publication volume and citation impact, it does not assess study quality or reproducibility, which are crucial for understanding the real-world applicability of findings. The inclusion of self-citations may also slightly overestimate some impact metrics, although prior research has shown this does not significantly alter overall bibliometric trends. Finally, the rapidly evolving nature of the field may limit the capture of emerging trends and technologies, warranting further investigation as the research landscape continues to develop.

## CRediT authorship contribution statement

**Caixia Tan:** Writing – original draft, Visualization, Validation, Software, Methodology, Formal analysis, Data curation, Conceptualization. **Jie Wang:** Methodology. **Anhua Wu:** Writing – review & editing, Project administration, Funding acquisition. **Chunhui Li:** Writing – review & editing, Project administration, Funding acquisition.

## Ethics approval and consent to participate

Not Applicable.

## Consent for publication

Not applicable.

## Funding

This work was supported by the 10.13039/501100012166National Key Research and Development Program of China [No. 2022YFC2009801; No. 2022YFC2009805], the 10.13039/501100004735Natural Science Foundation of Hunan Province [No. 2025JJ50648; 2026JJ82452], the Project program of National Clinical Research Center for Geriatric Disorders (Xiangya Hospital)[No. 2021KFJJ05], Innovation and Technology Commercialization Fund of 10.13039/501100011790Xiangya Hospital, Central South University [No. 2024ZHJJ06], and Scientific and Technological Personnel Lifting Project in Hunan Province [No. 2023 TJ-Z11].

## Declaration of competing interest

The authors declare that they have no known competing financial interests or personal relationships that could have appeared to influence the work reported in this paper.

## Data Availability

The original contributions presented in the study are included in the article/Supplementary Material, further inquiries can be directed to the corresponding author.
