# Supplementary material for: Mapping the global landscape of biofilm-associated antimicrobial resistance (1992–2025)
Source: Biofilm. 2026 Mar 10;11:100358. doi: 10.1016/j.bioflm.2026.100358 (PMC13010984; doi:10.1016/j.bioflm.2026.100358)
Supplement: Multimedia component 1 [file mmc1.docx]

**Table S1.** Detailed searching terms used in Web of Science Core Collection

| Concept group | Search terms (synonyms/variants) | Operator |
| --- | --- | --- |
| Antimicrobial resistance | "multidrug resistant*" OR "multi-drug resistant*" OR "multidrug-resistant*" OR "MDR" OR "multidrug resistance" OR "multi-drug resistance" OR "multidrug-resistance" OR "antibiotic* resistance" OR "drug-resistant bacteria" OR "drug-resistant bacterium" OR "antimicrobial resistance" OR "AMR" OR "antifungal resistance" OR "antifungal drug resistance" OR "drug-resistant fungi" OR "drug-resistant fungus" | OR |
| Biofilm | biofilm OR biofilms | OR |
| Combination | antimicrobial resistance AND Biofilm | AND |

**Table S2.** Screening and exclusion criteria for retrieved publications

| Exclusion Category | Definition | Example(s) | Number of Papers Excluded |
| --- | --- | --- | --- |
| Publication Time | Final publication date after December 31, 2025 | Articles with final publication dates in 2026 or later, post-print versions published after 2025, etc. | 40 |
| Document Type​ | Non-original research or review article | Meeting Abstract, Editorial Material, Letter, Early Access, Correction, News Item, Book Chapters, Proceeding Paper, Reprint, Withdrawn Publication, Item Withdrawal, etc. | 571 |
| Language​ | Publications not written in English | Articles in Chinese, Russian, Spanish, etc. | 94 |
| Duplicates | Duplicate records identified by CiteSpace | None detected | 0 |
| Total |  |  | 705 |

**Table S3**.Top 10 productive countries (above 5 papers) ranked by total publications or by average citations per article

| Ranked | Country* | Total publications | TC | AC | Country**#** | Total publications | TC | AC |
| --- | --- | --- | --- | --- | --- | --- | --- | --- |
| 1 | Denmark | 139 | 18908 | 136.028777 | China | 3407 | 80940 | 23.75697094 |
| 2 | Canada | 377 | 27242 | 72.25994695 | USA | 2198 | 157251 | 71.54276615 |
| 3 | USA | 2198 | 157251 | 71.54276615 | India | 1699 | 40951 | 24.10300177 |
| 4 | Ireland | 99 | 6255 | 63.18181818 | Brazil | 721 | 14648 | 20.31622746 |
| 5 | Switzerland | 95 | 5778 | 60.82105263 | Italy | 675 | 24526 | 36.33481481 |
| 6 | Netherlands | 118 | 6750 | 57.20338983 | Iran | 608 | 12782 | 21.02302632 |
| 7 | New zealand | 42 | 2397 | 57.07142857 | UK | 558 | 29551 | 52.95878136 |
| 8 | Belgium | 123 | 6616 | 53.78861789 | South korea | 480 | 11534 | 24.02916667 |
| 9 | UK | 558 | 29551 | 52.95878136 | Egypt | 452 | 8286 | 18.33185841 |
| 10 | Israel | 96 | 4462 | 46.47916667 | Spain | 435 | 17527 | 40.29195402 |
| Country*: country ranked by total publications; TC: total citations; AC: average citations; Country**#**: country ranked by AC; | | | | | | | | |

**Table S4**.Top 10 productive institutions (above 5 papers) ranked by total publications or by average citations per article

| Ranked | Institution* | | TC | Total Publications | AC | Institution# | TC | Total_ Publications | AC |
| --- | --- | --- | --- | --- | --- | --- | --- | --- | --- |
| 1 | Chinese Acad Sci | | 4965 | 129 | 44.00 | Allegheny Singer Res Inst | 6559 | 5 | 1311.80 |
|  | Univ Porto | | 5047 | 104 | 48.53 | Dartmouth Med Sch | 3941 | 6 | 656.83 |
| 3 | Zhejiang Univ | | 3173 | 95 | 33.40 | Rigshospitalet | 4186 | 7 | 598.00 |
| 4 | King Saud Univ | | 1452 | 66 | 22.00 | Univ Hosp Cleveland | 2531 | 5 | 506.20 |
| 5 | Alagappa Univ | | 2959 | 65 | 45.52 | Montana State Univ | 8784 | 24 | 366.00 |
| 6 | Islamic Azad Univ | | 669 | 65 | 10.29 | Tufts Univ | 2745 | 8 | 343.13 |
| 7 | Univ Minho | | 3766 | 63 | 59.78 | Univ Texas | 2313 | 8 | 289.13 |
| 8 | Zagazig Univ | | 1032 | 63 | 16.38 | Suny Binghamton | 3707 | 13 | 285.15 |
| 9 | Univ Copenhagen | | 7534 | 62 | 121.52 | Augusta Univ | 1394 | 5 | 278.80 |
| 10 | Sichuan Univ | | 2034 | 62 | 32.81 | Dalhousie Univ | 1545 | 6 | 257.50 |
|  | |  |  |  |  |  |  |  |  |

**Table S5**. Top 20 productive journals (above 5 papers) ranked by total publications or by average citations per article

| Ranked | Journal* | TC | Total Publications | AC | Journal# | TC | Total Publications | AC |
| --- | --- | --- | --- | --- | --- | --- | --- | --- |
| 1 | *Frontiers in Microbiology* | 30673 | 755 | 40.62649007 | *Nature Reviews Microbiology* | 20020 | 23 | 870.4347826 |
| 2 | *Antibiotics* | 13897 | 624 | 22.27083333 | *The Lancet Infectious Diseases* | 2539 | 5 | 507.8 |
| 3 | *Microbial Pathogenesis* | 8225 | 412 | 19.96359223 | *Nature* | 2380 | 5 | 476 |
| 4 | *Scientific Reports* | 9001 | 345 | 26.08985507 | *Trends in Microbiology* | 10554 | 25 | 422.16 |
| 5 | *Microorganisms* | 6958 | 330 | 21.08484848 | *Clinical Microbiology Reviews* | 4032 | 13 | 310.1538462 |
| 6 | *International Journal of Molecular Sciences* | 8052 | 303 | 26.57425743 | *Biotechnology Advances* | 1831 | 7 | 261.5714286 |
| 7 | *Antimicrobial Agents and Chemotherapy* | 20449 | 291 | 70.27147766 | *FEMS Microbiology Reviews* | 5755 | 26 | 221.3461538 |
| 8 | *PLOS ONE* | 10364 | 280 | 37.01428571 | *Genome Biology* | 953 | 5 | 190.6 |
| 9 | *Frontiers in Cellular and Infection Microbiology* | 8816 | 261 | 33.77777778 | *Drugs* | 1480 | 8 | 185 |
| 10 | *BMC Microbiology* | 5133 | 223 | 23.01793722 | *British Journal of Pharmacology* | 771 | 5 | 154.2 |
| 11 | *Microbiology Spectrum* | 2291 | 222 | 10.31981982 | *Advanced Drug Delivery Reviews* | 3074 | 20 | 153.7 |
| 12 | *Molecules* | 7059 | 181 | 39 | *Microbiology and Molecular Biology Reviews* | 899 | 6 | 149.8333333 |
| 13 | *Pathogens* | 3058 | 154 | 19.85714286 | *Drug Resistance Updates* | 1039 | 7 | 148.4285714 |
| 14 | *Journal of Applied Microbiology* | 5460 | 151 | 36.1589404 | *Proceedings of the National Academy of Sciences of the United States of America* | 6074 | 44 | 138.0454545 |
| 15 | *Applied and Environmental Microbiology* | 6114 | 138 | 44.30434783 | *Current Opinion in Microbiology* | 2197 | 17 | 129.2352941 |
| 16 | *Science of the Total Environment* | 5155 | 133 | 38.7593985 | *Science Translational Medicine* | 742 | 6 | 123.6666667 |
| 17 | *Infection and Drug Resistance* | 3383 | 132 | 25.62878788 | *Journal of Dental Research* | 1656 | 14 | 118.2857143 |
| 18 | *Pharmaceutics* | 2440 | 121 | 20.16528926 | *Biomaterials* | 4825 | 41 | 117.6829268 |
| 19 | *Archives of Microbiology* | 1681 | 121 | 13.89256198 | *Natural Product Reports* | 585 | 5 | 117 |
| 20 | *Current Microbiology* | 1135 | 118 | 9.618644068 | *FEMS Yeast Research* | 918 | 8 | 114.75 |

Journal*: journal ranked by total publications; TC: total citations; AC: average citations; Journal#: journal ranked by AC;


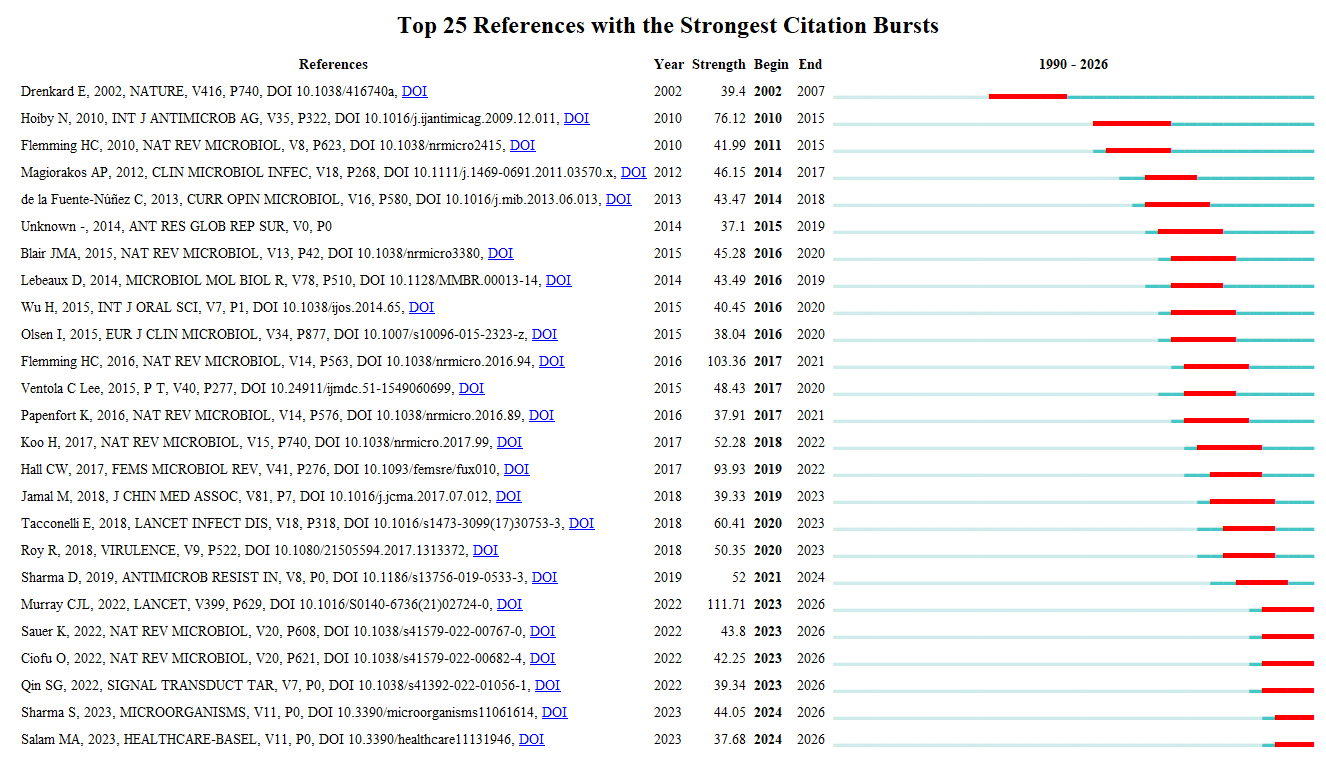


**Fig S1.** The top 25 co-cited references with the strongest citation bursts.
